# Supplementary figures and images for: Redundancy and Specificity of Type VI Secretion vgrG Loci in Antibacterial Activity of Agrobacterium tumefaciens 1D1609 Strain
Source: Front Microbiol. 2020 Jan 14;10:3004. doi: 10.3389/fmicb.2019.03004 (PMC6971182; doi:10.3389/fmicb.2019.03004)

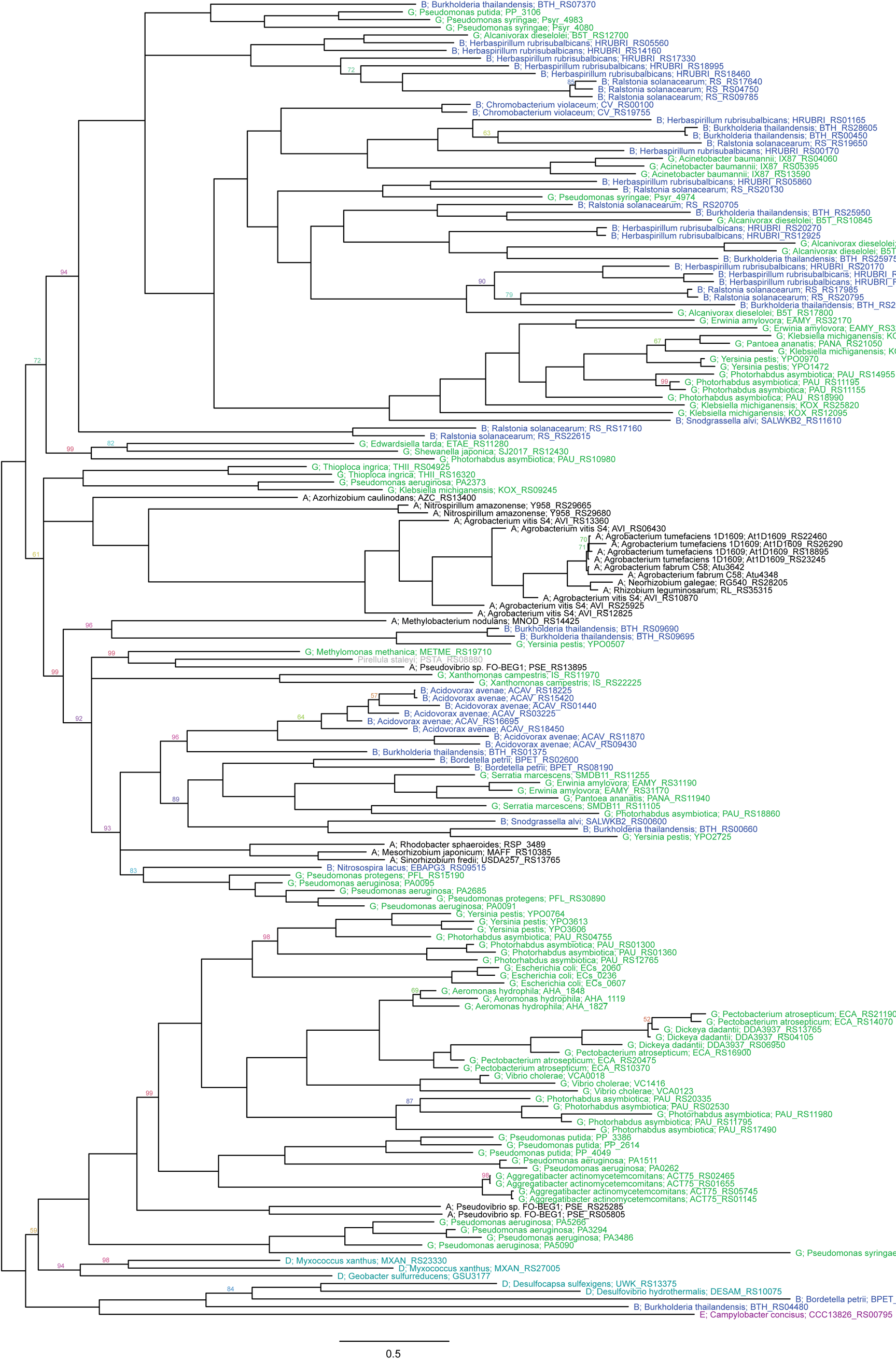

Supplement: FIGURE S1 — Bayesian phylogeny of the VgrG homologs identified in the representative Proteobacteria genomes presented in Figure 1. The color coding is based on the class: Alphaproteobacteria (black), Betaproteobacteria (blue), Deltaproteobacteria (cyan), Epsilonproteobacteria (purple) and Gammaproteobacteria (green). Numbers on the branches indicate the support levels based on posterior probabilities, only values > 60% were shown. [file Image_1.TIF]

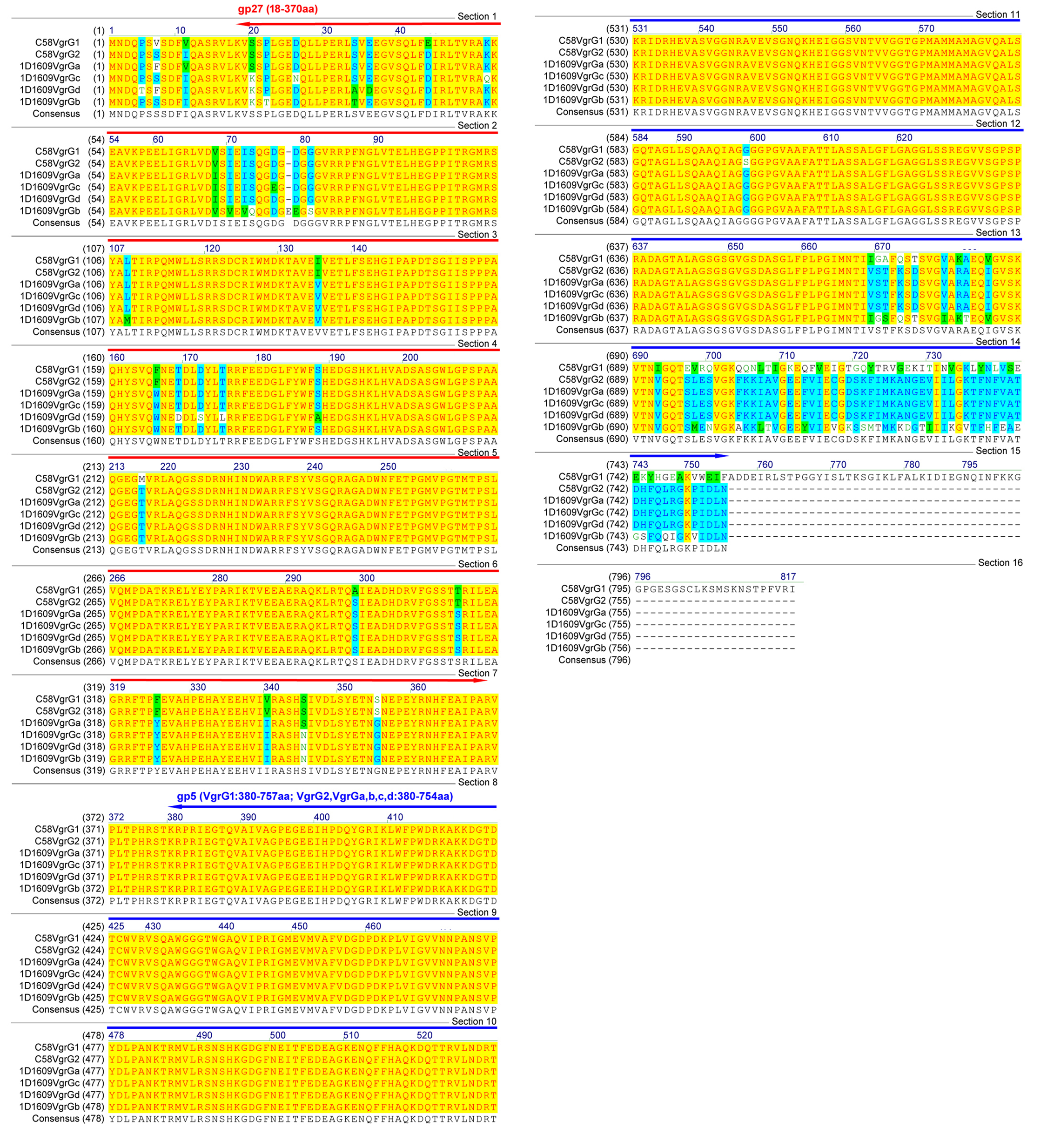

Supplement: FIGURE S2 — Multiple sequence alignment of VgrG amino acid sequences of 1D1609 and C58. All VgrG homologs were aligned and conserved amino acid residues are shaded in yellow and variable residues are in blue or green, while the C-terminal extension of C58 VgrG1 not homologous to the other VgrGs is unshaded. The red and blue lines indicate the predicted gp27 and gp5 domains, respectively. The amino acid residue number is indicated. [file Image_2.TIF]

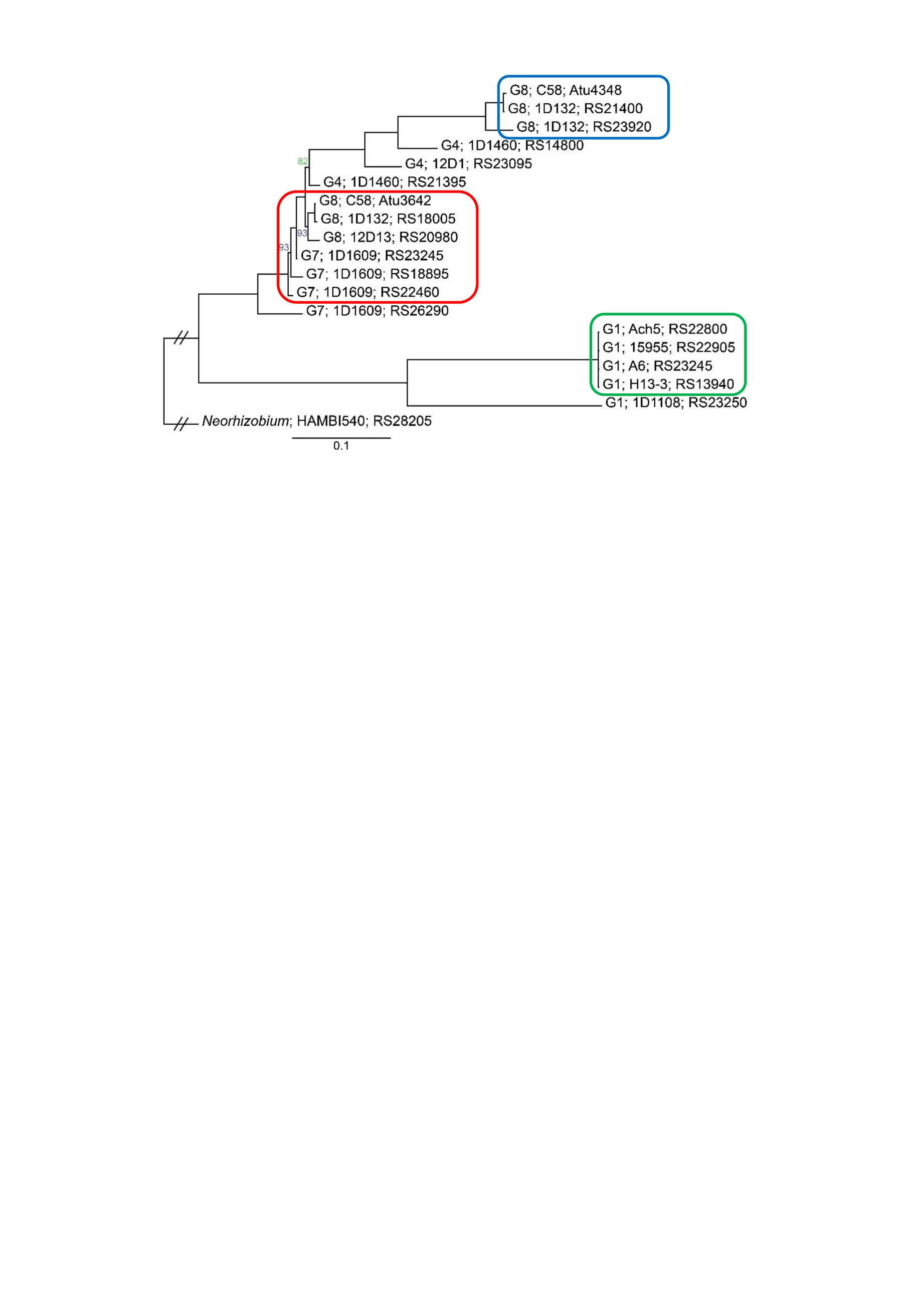

Supplement: FIGURE S3 — VgrG tree of the 11 A. tumefaciens strains. All VgrG homologs in these 11 genomes were extracted for inferring the gene tree. The VgrG orthologs share ≥ 95% identity in amino acid sequence were highlighted. The genomospecies and strain name for each VgrG indicated by a locus tag are shown. [file Image_3.TIF]

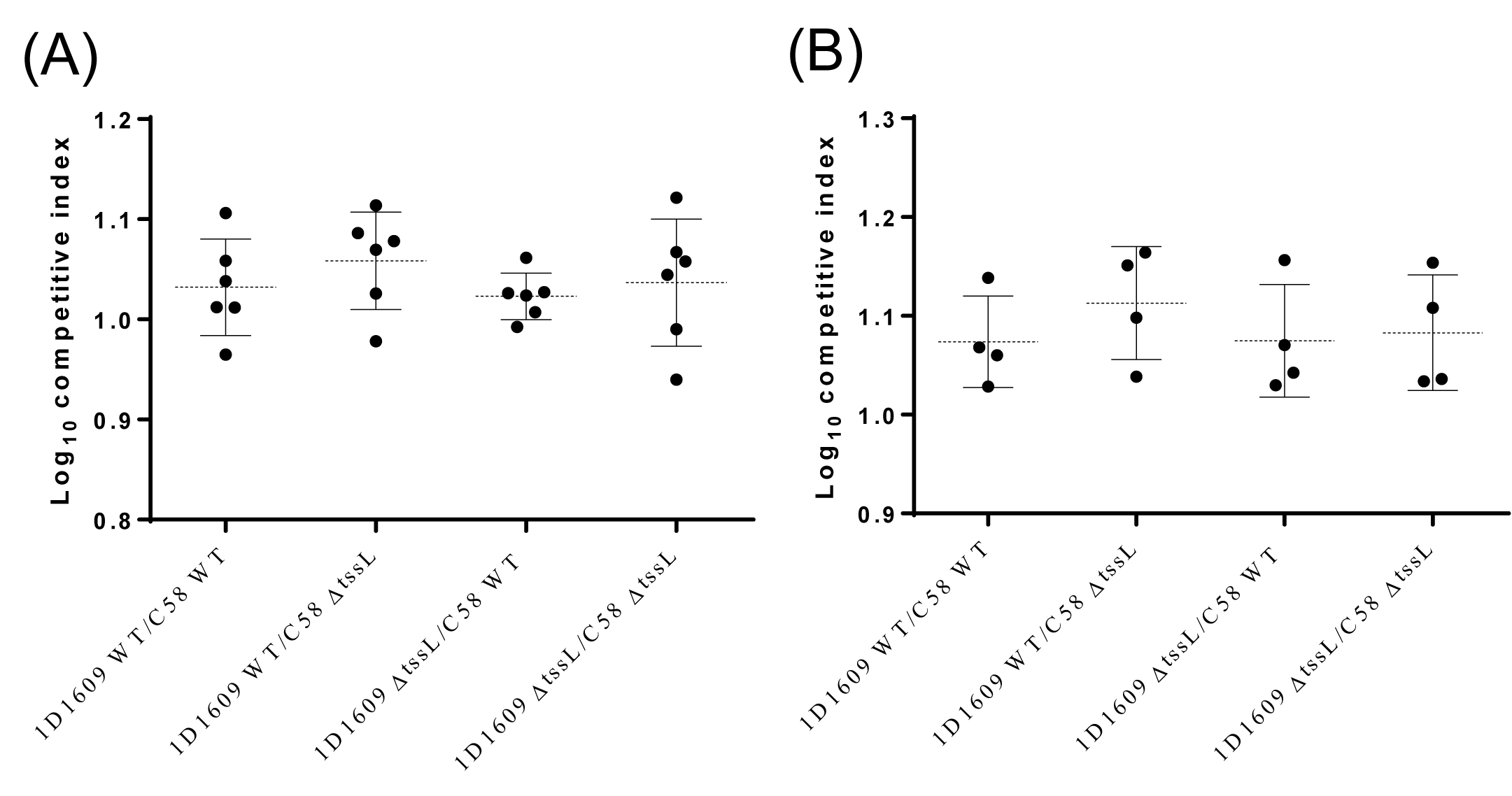

Supplement: FIGURE S4 — In planta competition experiments between C58 and 1D1609 at 1:1 ratio. A. tumefaciens strain C58 harboring gentamicin resistance-conferring pRL662 and strain 1D1609 which was selected in spectinomycin plate were mixed at 1:1 ratio and infiltrated into leaves of 6- to 7-week-old Nicotiana benthamiana (A) and Medicago truncatula (B). The competition outcome was quantified at 0 and 24 h post-infection by counting cfu on LB plates with appropriate antibiotics. Data are mean ± SEM, with each data point indicating the competitive index of 4–6 biological replicates from a total of 2–3 independent experiments. No statistical difference (P > 0.05) could be detected among different samples as determined by Tukey’s HSD test. [file Image_4.TIF]

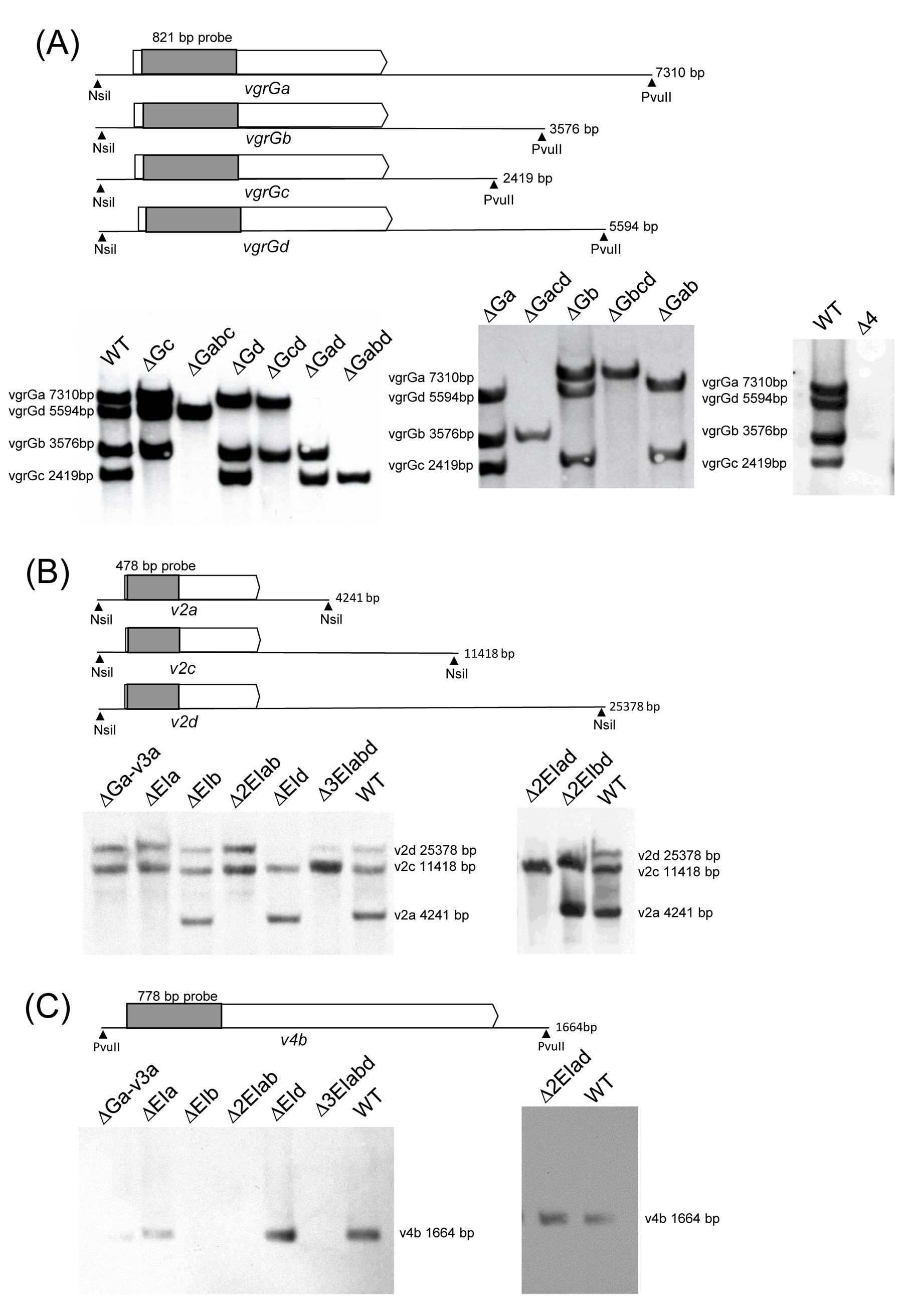

Supplement: FIGURE S5 — Southern blot analysis of mutants generated in this study. Schematic diagram showing probes used for Southern blotting, restriction enzyme cleavage sites and expected sizes. The genomic DNA is hybridized with (A) 821-bp vgrG probe with homology to all four vgrG genes (vgrGabcd) was used to confirm vgrG mutants; (B) 478-bp v2 probe with homology to v2acd was used to confirm v2a, v2c, and v2d mutant and (C) 778-bp v4b probe was used to confirm v4b mutant. Each lane contains 30 μg of genomic DNA digested with NsiI only or combined with PvuII. The expected size (in bp) is indicated on the side of the blot. [file Image_5.TIF]
