# Supplementary material for: Redundancy and Specificity of Type VI Secretion vgrG Loci in Antibacterial Activity of Agrobacterium tumefaciens 1D1609 Strain
Source: Front Microbiol. 2020 Jan 14;10:3004. doi: 10.3389/fmicb.2019.03004 (PMC6971182; doi:10.3389/fmicb.2019.03004)
Supplement: TABLE S4 — Bacterial strains and plasmids. [file Table_4.doc]

**Table S4.** Bacterial strains and plasmids

| **Strains** | **Relevant characteristics** | **Reference/ Source** | **EML No.** |
| --- | --- | --- | --- |
| ***E. coli*** |  |  |  |
| DH10B  DH10B (S17-1)  BL21(DE3) | Host of plasmid for cloning  Host for conjugation  Host of plasmid for expression | Lab stock  Lab stock  Lab stock | EML455  EML124  EML117 |
| ***A. tumefaciens C58*** |  |  |  |
| WT | wild type virulent strain (pTiC58,pAtC58) | Eugene Nester, University of Washington, USA | EML530 |
| ∆*tssL* | *tssL* deletion mutant | (1) | EML1073 |
| ∆*vgrG*1,2 | *vgrG1* and *vgrG2* deletion mutant | (2) | EML1289 |
| ∆*tdei*∆*G1*∆*G2op* | *tde1-tdi1, vgrG1,* and *vgrG2* operon deletion mutant | (3) | EML5130 |
| ***A. tumefaciens 1D1609*** |  |  |  |
| WT | Alfalfa isolate, virulent strain containing pTi1D1609 | Clarence Kado, UC Davis (4) | EML304 |
| ∆*tssL* | *tssL* in-frame deletion mutant | (5) | EML4718 |
| ∆*Ga* | *vgrGa* in-frame deletion mutant | This study | EML5297 |
| ∆*Gb* | *vgrGb* in-frame deletion mutant | This study | EML4729 |
| ∆*Gc* | *vgrGc* in-frame deletion mutant | This study | EML4731 |
| ∆*Gd* | *vgrGd* in-frame deletion mutant | This study | EML4734 |
| ∆G*ab* | *vgrGa* and *vgrGb* double deletion mutant | This study | EML5303 |
| ∆G*cd* | *vgrGc* and *vgrGd* deletion mutant | This study | EML4735 |
| ∆G*ad* | *vgrGa* and *vgrGd* deletion mutant | This study | EML5327 |
| ∆G*bcd* | *vgrGb*, *vgrGc* and *vgrGd* deletion mutant | This study | EML4739 |
| ∆*Gacd* | *vgrGa*, *vgrGc* and *vgrGd* deletion mutant | This study | EML5533 |
| ∆*Gabd* | *vgrGa*, *vgrGb* and *vgrGd* deletion mutant | This study | EML5518 |
| ∆*Gabc* | *vgrGa*, *vgrGb* and *vgrGc* deletion mutant | This study | EML5347 |
| ∆*Gabcd* (∆*4*) | *vgrGa*, *vgrGb*, *vgrGc* and *vgrGd* deletion mutant | This study | EML4746 |
| ∆*v2av3a* (∆EIa) | Deletion of *vgrGa*-associated effector-immunity pair | This study | EML5294 |
| ∆*v4bv5b* (∆EIb) | Deletion of *vgrGb*-associated effector-immunity pair | This study | EML5311 |
| ∆*vgrGc-v3c* (∆Gc-v3c) | Deletion of *vgrGc* loci including *vgrG*, chaperone and associated effector-immunity pair | This study | EML5538 |
| ∆*v2dv3d* (∆EId) | Deletion of *vgrGd*-associated effector-immunity pair | This study | EML5350 |
| ∆2EIab | Deletion of *vgrGa*-associated and *vgrGb*-associated effector-immunity pairs | This study | EML5316 |
| ∆2EIad | Deletion of *vgrGa-*associated and *vgrGd*-associated effector-immunity pairs | This study | EML5529 |
| ∆3EIabd | Deletion of *vgrGa*-, *vgrGb*- and *vgrGd*-associated effector-immunity pairs | This study | EML5515 |
| ∆4EIabcd (∆3EIabd∆Gc-v3c) | Deletion of *vgrGa*-, vgrGb- and *vgrGd*-associated effector-immunity pairs and whole *vgrGc* cluster (∆*vgrGc-v3c*) | This study | EML5540 |
|  |  |  |  |
| **Plasmids** |  |  |  |
| pJQ200KS | Gm^R^, suicide plasmid containing Gm^R^ and *sacB* gene for selection of double crossover | (6) | EML121 |
| pRL662 | Gm^R^, non-transferable broad host range vector derived from pBBR1MCS-2 | (7) | EML315 |
| pRLBla | Gm^R^ vector derived from pRL662, the *beta-lactamase* fragment containing Amp^R^ cassette from pBBR1-GFP was cloned into pRL662 | This study | EML4740 |
| pET22b(+) | Amp^R^, *E. coli* overexpression vector | Novagen | EML188 |
| pJN105 | Gm^R^, arabinose-inducible gene expression vector | (5) | EML4220 |
| pTrc200 | Sp^R^, pVS1 origin *lacIq*, *trc* promoter expression vector | (8) | EML904 |
| pTrc200-Gm | Gm^R^, *spectinomycin* resistance gene in pTrC200 was disrupted and replaced with *gentamicin* resistance gene | This study | EML5552 |
| pJQ200KS-*tssL* | Gm^R^, plasmid to generate *tssL* mutant | (5) | EML4705 |
| pJQ200KS-*Ga* | Gm^R^, plasmid to generate *vgrGa* mutant and quadruple *vgrG* mutant (∆*vgrGabcd*) | This study | EML4721 |
| pJQ200KS-*Gb* | Gm^R^, plasmid to generate *vgrGb* mutant, double *vgrG* mutant (∆*vgrGab*) and triple vgrG mutant (∆*vgrGbcd*) | This study | EML4723 |
| pJQ200KS-*Gc* | Gm^R^, plasmid to generate *vgrGc* mutant and triple *vgrG* mutant (∆*vgrGabc*) | This study | EML4725 |
| pJQ200KS-*Gd* | Gm^R^, plasmid to generate *vgrGd* mutant and double *vgrG* mutant (∆*vgrGcd* and ∆*vgrGad*) | This study | EML4733 |
| pRL662-TssL | Gm^R^, pRL662 constitutively expressing TssL | (5) | EML4708 |
| pRL662-VgrG1^C58^ | Gm^R^, pRL662 constitutively expressing C58 VgrG1 | (2) | EML1422 |
| pRL662-VgrG2^C58^ | Gm^R^, pRL662 constitutively expressing C58 VgrG2 | (2) | EML1419 |
| pRLBla-Ga | Gm^R^, Amp^R^ pRLBla constitutively expressing VgrGa | This study | EML4741 |
| pRLBla-Gb | Gm^R^, Amp^R^ pRLBla constitutively expressing VgrGb | This study | EML4751 |
| pRLBla-Gc | Gm^R^, Amp^R^ pRLBla constitutively expressing VgrGc | This study | EML4742 |
| pRLBla-Gd | Gm^R^, Amp^R^ pRLBla constitutively expressing VgrGd | This study | EML4750 |
| pJN-V2a | Gm^R^, pJN expressing *vgrGa*-associated effector | This study | EML5272 |
| pTrc-V3a | Sp^R^, pTrc200 expressing the *vgrGa*-associated immunity gene | This study | EML5274 |
| pJN-V2a (H385A) | Gm^R^, pJN expressing *vgrGa*-associated effector with amino acid substitution (H385A) | This study | EML5298 |
| pJN-V2a (H386A) | Gm^R^, pJN expressing *vgrGa*-associated effector with amino acid substitution (H386A) | This study | EML5300 |
| pJQ200KS-*v2av3a* | Gm^R^, plasmid to generate deletion of *vgrGa*-associated effector-immunity pair *v2a* and *v3a* | This study | EML5283 |
| pJQ200KS –*v4bv5b* | Gm^R^, plasmid to generate deletion of *vgrGb*-associated effector-immunity pair *v4b* and *v5b* | This study | EML5307 |
| pJQ200KS –*vgrGc-v3c* | Gm^R^, plasmid to generate deletion of *vgrGc* loci – vgrG, chaperone *v1c* and associated effector-immunity pair *v2c* and *v3c* | This study | EML5537 |
| pJQ200KS -*v2v3d* | Gm^R^, plasmid to generate deletion of *vgrGd*-associated effector-immunity pair *v2d* and *v3d* | This study | EML5340 |
| pTrc-Atu3641-Tde2^M^ | Sp^R^, pTrc200 expressing *atu3641* and *tde2* catalytic site mutant | (9) | EML4794 |

**References**

1. Ma LS, Lin JS, Lai EM. An IcmF family protein, ImpLM, is an integral inner membrane protein interacting with ImpKL, and its walker a motif is required for type VI secretion system-mediated Hcp secretion in Agrobacterium tumefaciens. J Bacteriol. 2009;191(13):4316-29.

2. Lin JS, Ma LS, Lai EM. Systematic Dissection of the Agrobacterium Type VI Secretion System Reveals Machinery and Secreted Components for Subcomplex Formation. PLoS One. 2013;8(7):e67647.

3. Wu C-F, Lien Y-W, Bondage D, Lin J-S, Pilhofer M, Shih Y-L, Chang JH, Lai E-M. 2019. Effector loading onto the VgrG carrier activates type VI secretion system assembly. EMBO Rep (2019)e47961. https://doi.org/10.15252/embr.201947961

4. Palumbo JD, Phillips DA, Kado CI. Characterization of a new Agrobacterium tumefaciens strain from alfalfa. Archives of microbiology. 1998;169(5):381-6.

5. Wu CF, Santos MNM, Cho ST, Chang HH, Tsai YM, Smith DA, Kuo CH*, Chang JH*, Lai EM* (2019) Plant pathogenic Agrobacterium tumefaciens strains have diverse type VI effector-immunity pairs and vary in in planta competitiveness. Molecular Plant-Microbe Interactions 32: 961-971. DOI: 10.1094/MPMI-01-19-0021-R 6. Quandt J, Hynes MF. Versatile suicide vectors which allow direct selection for gene replacement in gram-negative bacteria. Gene. 1993;127(1):15-21.

7. Vergunst AC, Schrammeijer B, den Dulk-Ras A, de Vlaam CM, Regensburg-Tuink TJ, Hooykaas PJ. VirB/D4-dependent protein translocation from Agrobacterium into plant cells. Science (New York, NY). 2000;290(5493):979-82.

8. Schmidt-Eisenlohr H, Domke N, Baron C. TraC of IncN Plasmid pKM101 Associates with Membranes and Extracellular High-Molecular-Weight Structures in<em>Escherichia coli</em>. Journal of Bacteriology. 1999;181(18):5563-71.

9. Bondage DD, Lin J-S, Ma L-S, Kuo C-H, Lai E-M. VgrG C terminus confers the type VI effector transport specificity and is required for binding with PAAR and adaptor–effector complex. Proceedings of the National Academy of Sciences. 2016;113(27):E3931-E40.
