# Supplementary material for: Redundancy and Specificity of Type VI Secretion vgrG Loci in Antibacterial Activity of Agrobacterium tumefaciens 1D1609 Strain
Source: Front Microbiol. 2020 Jan 14;10:3004. doi: 10.3389/fmicb.2019.03004 (PMC6971182; doi:10.3389/fmicb.2019.03004)
Supplement: TABLE S5 — Primers used in this study. [file Table_5.DOC]

| **Table S5**. Primers used in this study | |
| --- | --- |
|  |  |
| Plasmid | Primer Sequence |
|  |  |
| pJQ200KS-*vgrGa* | 5'-GCTCTAGAACCTGCGCCATTCGCGTCAGC-3' |
|  | 5'-CGGGATCCCTGGTCGTTCATGGGATACTCCTA-3' |
|  | 5'-CGGGATCCGATCTGAACTGACGGAGGCCG-3' |
|  | 5'-TCCCCCCGGGAGCGTTGCGCCAGCATATGGC-3' |
|  |  |
| pJQ200KS-*vgrGb* | 5'-GCTCTAGATGTCACCGGCAGTGCCCTCAGCC-3' |
|  | 5'-CGGGATCCCTGGTCATTCATTGCGAACCTCGA-3' |
|  | 5'-CGGGATCCGATCTCAACTGATCGGGTTCGTGG-3' |
|  | 5'-TCCCCCCGGGCAATGCCTTCAGCCAGATCG-3' |
|  |  |
| pJQ200KS-*vgrGc* | 5'-GCTCTAGACTGTCCCTCATTCGTCATCAGGG-3' |
|  | 5'-CGGGATCCCTGGTCATTCATTGCGAACCTCGA-3' |
|  | 5'-CGGGATCCCTGAACTGACGGAGGCCG-3' |
|  | 5'-TCCCCCCGGGGCGCTCCACGTCAATCGG-3' |
|  |  |
| pJQ200KS-*vgrGd* | 5'-GCTCTAGACGCAAATGGCTGAGGGCAC-3' |
|  | 5'-CGGGATCCGGGCTGGTCATTCATTGCGAACCTC-3' |
|  | 5'-CGGGATCCGATCTCAACTGATCGGGTTCGTGG-3' |
|  | 5'-TCCCCCCGGGCAATGCCTTCAGCCAGATCGG -3' |
|  |  |
| pRL662-VgrGa | 5'-CCGCTCGAGGAAACGATCTGGTTCT-3' |
|  | 5'-GCTCTAGAAGGCGGTTCTGAAGTTCCAA-3' |
|  |  |
| pRL662-VgrGb | 5'-CCGCTCGAGCTGTCAGATTTCATCGATGGA-3' |
|  | 5'-GCTCTAGAGCAGGTTCGCAAATGGCGTG-3' |
|  |  |
| pRL662-VgrGc | 5'-CCGCTCGAGTCTGTCGAGATTTTGACAGC-3' |
|  | 5'-GCTCTAGACGTCAGTTCGATGCCGA-3' |
|  |  |
| pRL662-VgrGd | 5'-CCGCTCGAGGATTGCATTCTGTCGAGA-3' |
|  | 5'-GCTCTAGATCGGCATCGAACTGACGGA-3' |
|  |  |
| pJN-V2a | 5'-CCGGAATTCAGGAGGACAGCTATGAGCATCCCCCGCGACAATTAT-3' |
|  | 5'-GCTCTAGATCATGGTTTTCTGGTCGCTCCTTC-3' |
|  |  |
| pTrc-V3a | 5'-CATGCCATGGCCTGACGACCCAATATGCTGA-3' |
|  | 5'-GCTCTAGACGGTGCACATTGCTGCGATT-3' |
|  |  |
| pJN-V2a(H385A) | 5'-CCGGAATTCAGGAGGACAGCTATGAGCATCCCCCGCGACAATTAT-3' |
|  | 5'-TGGAACTATATG**CGC**AGCCTGCATGCC-3' |
|  | 5'-GGCATGCAGGCT**GCG**CATATAGTTCCA-3' |
|  | 5'-GCTCTAGATCATGGTTTTCTGGTCGCTCCTTCA-3' |
|  |  |
| pJN-V2a(H386A) | 5'-CCGGAATTCAGGAGGACAGCTATGAGCATCCCCCGCGACAATTAT-3' |
|  | 5'-GTCTGGAACTAT**CGC**GTGAGCCTGCATGCCGTA-3' |
|  | 5'-TACGGCATGCAGGCTCAC**GCG**ATAGTTCCAGAC -3' |
|  | 5'-GCTCTAGATCATGGTTTTCTGGTCGCTCCTTCA-3' |
|  |  |
| pJQ200KS-*v2av3a* | 5'-GCTCTAGATTCCGGAACTTATGACGACGGTTG-3' |
|  | 5'-CGGGATCCGGGGATcGCTCATGCTGTCTCC-3' |
|  | 5'-CGGGATCCCCAGATCTCTGAGTTGCGAGCTTC-3' |
|  | 5'-AACTGCAGACAGGGCTACATCTATGCACCG-3' |
|  |  |
| pJQ200KS-*v4bv5b* | 5'-TTTCTAGACATCTGTTGTTCGCGGCCGATG-3' |
|  | 5'-GCGGATCCATCACCCGCCATGTGAGGCCT-3' |
|  | 5'-GCGGATCCTTCGCTGCCCTGTGACCGTTCT-3' |
|  | 5'-GCCCCGGGGCAAGATGATTCCGGGAATTGAC-3' |
|  |  |
| pJQ200KS-*vgrGc-v3c* | 5'-TTTCTAGATCGGCGAGACGCACTTCAG-3' |
|  | 5'-GCGGATCCCTGGTCATTCATTGCGAACC-3' |
|  | 5'-GCGGATCCGCCAGGACTTGACCACCGTA-3' |
|  | 5'-AACTGCAGCATTGAAGCGTTCAATGGCCG-3' |
|  |  |
| pJQ200KS-*v2dv3d* | 5'-GCTCTAGAGAGCGCCGAAATCCGCTT-3' |
|  | 5'-CGGGATCCGATGCTCATGCTGTCTCCCTCTC-3' |
|  | 5'-CGGGATCCGCCAGAAAGCCATAGATTGTCG-3' |
|  | 5'-TCCCCCCGGGGCATCACCACACTCTCGAGCCT-3' |
|  |  |
| pRLBla-V2aV3a | 5'-CCGCTCGAG TTCTGACCTGGCGCATCAGGTTTC-3' |
|  | 5'-GCTCTAGA AAACACCAGCCGAGCTAACCGCACTC-3' |
|  |  |
| pRLBla-VgrGa | 5'-CCGCTCGAGGAAACGATCTGGTTCT-3' |
|  | 5'-GCTCTAGAAGGCGGTTCTGAAGTTCCAA-3' |
|  |  |
| *vgrG* probe | 5’-CCGCAAATGTGGCTTCTGTC-3’ |
|  | 5’-CCTTCGATGCGCGGGCGTTTTGTTG-3’ |
|  |  |
| *v2a, v2c* and *v2d* probe | 5’-GCGACAATTATATCGGAGAGCC-3’ |
|  | 5’-GATCATCGTCCTTGGGAGGATC-3’ |
|  |  |
| *v4b* probe | 5’-GTGATGAAGAGGTCCATCAGCGCAATG-3’ |
|  | 5’-GCATCGATATCGCTCAGTCGG-3’ |
|  |  |
|  |  |
| Restriction enzyme sites are underlined and mutations are in bold. | |
